# Ending Abusive Patient- and Family-Initiated Relationships in Alberta Nursing Practice: The Case for a Nurse-Specific Regulatory Standard

**DOI:** 10.3390/nursrep16070212

**Published:** 2026-06-25

**Authors:** Dawid Karczewski, Tomasz Karczewski, Mihaela Olsen

**Affiliations:** Cranston Ridge Medical Clinic, Calgary, AB T3M 3A9, Canada; tomasz@cranstonridgemedical.com (T.K.); mihaela@cranstonridgemedical.com (M.O.)

**Keywords:** nursing regulation, workplace violence, patient- and family-initiated abuse, nurse–patient relationship, abandonment, occupational safety, nurse practitioners, self-employed nursing practice, Alberta, professional regulation

## Abstract

**Background/Objectives:** Patient- and family-initiated abuse of nurses is widely recognized as workplace violence, but it also raises a distinct professional-regulatory question: when may a nurse end or restrict an established therapeutic relationship without creating an allegation of abandonment or discriminatory denial of care? This perspective focuses on Alberta and examines whether the province’s nursing regulator provides a comprehensive, nurse-specific, all-registrant standard comparable in clarity to Alberta’s physician standard. The concern is not the absence of every form of protection but the absence of a clearly defined regulatory pathway for all Alberta RNs and NPs. **Methods:** Publicly available legal, occupational health and safety, regulatory, legal-risk and scholarly materials were purposively selected where they addressed relationship termination, discontinuing or declining care, workplace violence, immediate safety risk, abandonment, documentation, continuity of care or patient safeguards. **Results:** The College of Physicians and Surgeons of Alberta standard provides a clearly defined regulatory pathway, including immediate discharge where a patient poses a safety risk, is abusive or fails to respect professional boundaries. Alberta nursing materials contain important elements but do not yet constitute a dedicated Alberta RN/NP standard applicable across office-based, community, virtual, home care and independent nursing practice. Interprovincial nursing standards demonstrate feasibility, operational detail, emerging professional consensus and potential templates for policy transfer; however, they do not bind the CRNA or create an Alberta regulatory benchmark for complaint review. **Conclusions:** Alberta should adopt a nurse-specific standard for ending or restricting abusive patient- and family-initiated relationships. Such a standard should include ordinary and urgent safety pathways, prohibited grounds, documentation requirements, continuity safeguards, employer integration and practical templates. Nurse protection and patient protection are mutually reinforcing regulatory objectives.

## 1. Introduction

Patient- and family-initiated abuse of nurses is commonly described as a workplace-violence problem. That description is accurate but incomplete. After an abusive incident, the question is not only whether an employer should investigate, whether security should be called, or whether criminal law may apply. A second and more specific question arises for nursing regulation: when an established nurse–patient relationship has become abusive, threatening, harassing or unsafe, how may the nurse end or restrict that relationship while avoiding abandonment, discrimination and unsafe interruption of care?

Alberta is an important jurisdiction in which to ask this question. Physicians in Alberta have a dedicated College of Physicians and Surgeons of Alberta (CPSA) standard governing termination of the physician–patient relationship in office-based settings. This standard requires reasonable grounds and documentation, prohibits discriminatory termination, requires notice and continuity safeguards, and expressly permits immediate discharge where the patient poses a safety risk, is abusive, or fails to respect professional boundaries [1]. The CPSA’s companion advice provides further operational guidance. It states that a physician may discharge a patient immediately when the patient is physically or verbally abusive; threatening or violent toward the physician, staff members or other patients; and treats racist or discriminatory language toward physicians or staff as abusive and threatening [2].

The central concern of this article is legal–regulatory parity within the same province. Alberta nurses are regulated under Alberta’s professional framework, practise under Alberta occupational health and safety requirements, and serve the same public as Alberta physicians. If Alberta has already recognized, for physicians, that abusive patient conduct may justify immediate termination subject to safeguards, then Alberta nursing regulation should provide nurses with an equivalent, nurse-specific pathway. This is not an argument for arbitrary refusal of care. It is an argument for a clear standard that protects nurses from abuse while protecting patients from discrimination, retaliation and avoidable disruption of clinically necessary care.

The need for clarity has increased as nursing practice has expanded beyond hospital-based employment models. Registered nurses (RNs) and nurse practitioners (NPs) provide care in primary care, specialty clinics, community programs, home care, virtual platforms, public health, long-term care, occupational health, and private and self-employed practice. In these settings, abuse may occur within an ongoing relationship rather than during a single shift assignment. A nurse may be the sole clinician, the practice owner, the records custodian, the person receiving abusive electronic communications, and the professional expected to arrange continuity. The regulatory pathway must be sufficiently clear for that reality.

This perspective therefore proposes a dedicated Alberta nursing standard, titled for example ‘Ending or Restricting the Nurse-Patient Relationship in Office-Based, Community, Virtual, Home Care and Independent Practice.’ The proposed standard should be Alberta-first, because the regulatory gap is currently framed in Alberta law and Alberta professional practice. At the same time, the model is transferable: any nursing jurisdiction could adapt the same principles to its own legislation, human rights framework, regulator structure and health-system organization.

## 2. Scope, Approach and Source Selection

### 2.1. Jurisdictional Scope: Alberta-First, Not Alberta-Only

The primary recommendation in this manuscript is addressed to Alberta nursing regulation and to the College of Registered Nurses of Alberta (CRNA). This scope is intentionally limited to Alberta because the CRNA establishes the professional standards governing Alberta RNs and NPs within the province’s statutory framework. Accordingly, an authoritative benchmark for Alberta practice and regulatory assessment must be located in Alberta legislation, CRNA standards, or CRNA-adopted guidance with recognized professional effect [3].

The Alberta focus does not diminish the comparative value of standards from other jurisdictions. Such materials can demonstrate feasibility, supply operational detail, show convergent regulatory approaches and offer templates for policy transfer. Their function, however, differs from that of an Alberta standard: they can inform the CRNA’s policy design but cannot bind the Alberta regulator, govern complaints against Alberta registrants or independently authorize conduct that is inconsistent with Alberta requirements. Transferability therefore lies in adapting useful design features to Alberta’s legal and professional framework, not in treating an external standard as if it already resolved the Alberta gap.

### 2.2. Why the CPSA Standard Is the Central Comparator

A natural question is why this article compares Alberta nursing regulation with the CPSA standard rather than centering the analysis exclusively on nursing standards from other provinces. The answer turns on the type of comparison being made. The CPSA is used as the principal jurisdictional comparator because the problem concerns the professional protection available to Alberta nurses within Alberta. Nursing standards from other provinces are used as profession-specific design comparators. These two forms of comparison are complementary rather than competing.

The CPSA is not used because medicine is superior to nursing or because nursing should copy medicine uncritically. It is used because it provides the most directly relevant Alberta evidence that a termination pathway can be concise, enforceable and operational within the province. CPSA standards are enforceable under the Health Professions Act and are referenced in complaint management and discipline [1]. The termination standard operates in the same provincial health-service, human-rights and workplace-safety context and addresses the same operational event: ending an established relationship when abuse, safety risk or boundary violations make ongoing direct care untenable [1,2]. Ontario, British Columbia and Saskatchewan nursing materials, by contrast, offer closer profession-specific analogues for procedural design. The CPSA supplies the in-province regulatory precedent; the nursing comparators supply tested nursing approaches to documentation, continuity, resolution efforts and transfer. Both are necessary to the analysis.

### 2.3. Document Selection and Analytical Approach

This article is a narrative regulatory and policy perspective. It is not an empirical study, a systematic review, or jurisdiction-specific legal advice. Publicly accessible sources were purposively selected between May and June 2026 from official regulator websites, government legal and occupational health and safety sources, Canadian legal-risk resources for nurses, selected international regulatory or organizational frameworks, and peer-reviewed workplace-violence literature. Purposive selection was used to identify materials that directly addressed relationship termination; discontinuing or declining care; immediate safety risk; patient-, family- or visitor-initiated abuse; abandonment; continuity of care; documentation; professional boundaries; human rights safeguards; workplace violence; or employer prevention duties.

Materials were excluded where they were archived, superseded, inaccessible, not relevant to regulated nursing or comparable regulated health practice, or focused solely on abuse committed by clinicians against patients without addressing abuse directed toward clinicians. The CPSA was deliberately included as a physician regulator because the article’s central question is whether Alberta nursing regulation should provide a comparable Alberta pathway for nurses. Each document was mapped against seven questions: (1) whether it was Alberta-specific; (2) whether it applied to RNs, NPs or both; (3) whether it expressly permitted immediate termination or restriction for abuse or safety risk; (4) whether it prohibited discriminatory or retaliatory termination; (5) whether it required documentation; (6) whether it required continuity safeguards; and (7) whether it provided concise and operationally detailed tools for office-based, community, virtual, home care and independent practice.

## 3. Regulatory Findings

### 3.1. The Alberta Physician Standard Provides a Clearly Defined Regulatory Pathway

The CPSA standard is concise and operationally detailed. It requires reasonable grounds and documentation before a physician terminates a relationship; prohibits termination based on protected grounds or other improper reasons; requires written notice, a timeline commensurate with the patient’s continuing care needs, interim care, follow-up for outstanding investigations and transfer of information; and allows for immediate discharge when the patient poses a safety risk to office staff, other patients or the physician; is abusive to the physician, staff or other patients; or fails to respect professional boundaries [1].

The importance of the CPSA model is not simply that it permits discharge. Its importance is that it regulates discharge. The standard prevents arbitrary or discriminatory dismissal while acknowledging that clinician and staff safety can justify immediate action. It does not force a physician to choose between tolerating abuse and risking an unsupported termination decision. This is the type of dual protection that nurses also require.

### 3.2. Alberta Nursing Materials Contain Building Blocks but Not an Equivalent All-Registrant Standard

Alberta nursing regulation already contains important foundations. The CRNA’s Practice Standards for Registrants are the minimum expectations for safe, competent and ethical RN and NP practice, guide decision-making, and are described by the CRNA as a legal reference for reasonable and prudent practice [3]. The CRNA’s Nurse Practitioner Frequently Asked Questions state that an NP should make reasonable efforts to address concerns before ending a relationship, document reasons, provide an appropriate timeline, support continuity of care, and avoid ending a relationship on discriminatory or improper grounds; the same FAQ also states that in situations involving threats, abuse or safety concerns, the relationship may be ended immediately [4]. The CRNA’s self-employed practice advice recognizes that RNs may provide professional services in self-employed or independent practice, and the CRNA’s documentation and unsafe-practice materials reinforce documentation, accountability, reporting and response to threats to safety [5,6,7].

These materials are valuable and should be acknowledged. They also demonstrate that Alberta nursing regulation is already moving toward recognition of the problem. However, they do not yet produce functional parity with the CPSA model. First, the clearest Alberta nursing language on immediate termination appears in an NP FAQ, not in a dedicated RN/NP standard that applies to all relevant registrants. Second, the materials are distributed across practice standards, FAQs, self-employed practice advice, documentation standards and unsafe-practice guidance, rather than integrated into one enforceable pathway. Third, the NP FAQ itself points readers to CPSA and CNPS resources [4], confirming that the most operational termination model remains external to nursing regulation. Fourth, self-employed and clinic-based RNs require a standard that speaks directly to their setting, records, privacy, continuity and safety responsibilities.

Taken together, Alberta nursing materials provide general professional standards, occupational safety protections, legal resources and NP-specific guidance. The remaining gap is narrower but still consequential: Alberta lacks a single nurse-specific, all-registrant standard that tells RNs and NPs, in terms as clear as the CPSA, when and how an abusive relationship may be restricted or ended, including immediately, and what safeguards are required.

### 3.3. Criminal Law, Occupational Safety Law and Human Rights Law Are Necessary but Insufficient

Canadian criminal law, Alberta occupational health and safety law and Alberta human rights law provide essential but distinct protections. Section 423.2 of the Criminal Code makes it an offence to intimidate a health professional to impede the performance of duties or to obstruct lawful access to health services [8]. Alberta’s Occupational Health and Safety Code requires employers to maintain violence and harassment prevention plans, investigate incidents, train workers and support workers reporting injury or adverse symptoms [9,10], while human rights law prohibits discrimination in services and employment on protected grounds [11]. These sources address intimidation, workplace hazards, employer duties and discrimination, but none explains how an Alberta nurse should end or restrict an established nurse–patient relationship after abuse while documenting the decision, preserving urgent care, managing outstanding tests, transferring records, involving the employer and avoiding abandonment. Their requirements should be integrated into, but cannot replace, a nurse-specific professional-regulatory pathway.

### 3.4. Canadian Nursing Comparators Demonstrate Feasibility and Inform Design

Several Canadian nursing regulators have operationalized discontinuing or declining care. The College of Nurses of Ontario (CNO) has an all-nurse practice standard addressing professionalism, communication, safety and continuity when care is discontinued or declined [12]. Its NP guidance is more stepwise: it identifies threatening or abusive behaviour, including sexualized or racist comments posing a risk of harm, as a possible basis for ending the NP–client relationship; directs reasonable efforts to resolve concerns where possible; requires employer or team consultation, written communication and documentation; and addresses essential services, transfer, records, outstanding tests and a reasonable transition time [13]. In those respects, the CNO NP guidance is a close nursing analogue and is comparable in part to the procedural detail of the CPSA model. It is not identical in regulatory form or immediacy: the CNO frames discontinuation around resolution efforts and transition safeguards, whereas the CPSA contains an express clause permitting immediate discharge for abuse, safety risk or boundary violations [1,13].

British Columbia and Saskatchewan provide additional nursing analogues. The BCCNM’s all-nurse duty-to-provide-care standard permits withdrawal from or refusal of care where providing care would place the nurse or client at an unacceptable level of risk, while retaining legal, professional and continuity obligations [14]. The BCCNM’s NP learning materials address ending the NP–client relationship and abandonment risk [15]. The College of Registered Nurses of Saskatchewan (CRNS) identifies threats or abuse by a client or family as a reason to end the NP–client relationship and states that where there is a genuine risk of harm, the NP may end the relationship immediately and need not engage directly with the client [16]. These NP-specific sources are particularly valuable because they are close professional analogues and show how nursing regulators can operationalize safety, documentation and continuity.

These comparators have four practical functions for Alberta reform: they provide proof of feasibility, examples of operational detail, evidence of convergent regulatory approaches and templates for policy transfer. Their value is therefore more than merely informative. Their limitation is jurisdictional, not conceptual, and is not a criticism of their quality. The CNO, BCCNM and CRNS can guide the design of a CRNA standard, but they do not govern Alberta registrants or complaints. An external standard may be persuasive in policy development and comparative analysis, but it cannot displace CRNA requirements or independently create authority for an Alberta registrant. The sources also vary in scope: the CNO’s general standard applies to all nurses but is principle-based, whereas the more detailed CNO, BCCNM and CRNS materials discussed here are partly NP-specific. This NP focus makes them close professional analogues, yet it does not resolve the need for an Alberta all-registrant RN/NP standard. The CPSA remains the central Alberta jurisdictional precedent, while interprovincial nursing standards supply tested nursing-specific design elements.

### 3.5. International Context

International materials also support the need for better protection, although they do not provide an Alberta solution. The NHS England’s updated Violence Prevention and Reduction Standard requires organizations to support staff to feel safe, report violence and abuse, and have confidence that action will be taken; it is organized around leadership, governance, collaboration, data, workforce, interventions and evaluation [17]. In Australia, the Nursing and Midwifery Board of Australia’s professional standards and code resources set out legal and professional behaviour and conduct expectations for nurses in all practice settings [18]. In New Zealand, the Nursing Council’s Code of Conduct identifies expected professional conduct and emphasizes public trust, professional boundaries, and respect [19]. In the United States, the American Nurses Association’s workplace-violence position statement emphasizes prevention, reporting, environmental controls, education and post-incident support [20].

These examples confirm that violence and safety are global nursing concerns. They also illustrate the difference between workplace-violence prevention and relationship-termination regulation. International frameworks generally emphasize safe systems and reporting and conduct expectations and employer controls. They do not give Alberta nurses an Alberta-specific, regulator-endorsed pathway for ending or restricting an abusive patient relationship. The proposed standard would therefore contribute to international nursing regulation by translating workplace-safety principles into a relationship-specific professional standard. Table 1 summarizes the comparator findings and their implications for the proposed Alberta nursing standard.

## 4. Abuse, Safety, Patient Rights and Regulatory Risk

### 4.1. Patient- and Family-Initiated Abuse Is a Safety and Care-Quality Issue

Nursing depends on a therapeutic relationship grounded in trust, respect, professional boundaries, empathy and clinical judgment. Abuse from a patient, family member or visitor can damage those conditions. Threats, stalking, repeated verbal abuse, sexual harassment, discriminatory slurs, intimidation, boundary violations and aggressive electronic communications do not merely affect the nurse as a worker. They may also make safe assessment, objective clinical judgment, effective communication and compassionate care impossible.

Workplace-violence research supports the seriousness of the problem. A systematic review and meta-analysis by Liu et al. reported substantial worldwide exposure of health care workers to workplace violence, including physical and non-physical violence [21]. An umbrella review by Rossi et al. found workplace violence to be widespread across health care settings and emphasized prevention and policy interventions [22]. A Canadian framework article by Schulz-Quach et al. described workplace violence in health care as a global phenomenon requiring better measurement and coordinated system response [23]. The Canadian Federation of Nurses Unions reported in 2026 that 95% of respondents in a national survey of 4703 nurses had experienced some form of workplace violence or harassment in the previous year, while only 46% reported incidents of violence [24]. A 2025 Canadian white paper similarly described violence against nurses as a major system-level problem requiring enforcement, prevention and accountability [25].

Underreporting adds to the regulatory problem. Spencer et al.’s systematic review of nurses’ rationale for underreporting patient- and visitor-perpetrated workplace violence identified normalization, perceived futility, fear of consequences and organizational barriers among reasons why nurses do not report [26]. A clear termination-or-restriction standard would not eliminate underreporting by itself. However, it would make abusive conduct more professionally visible. It would give nurses a recognized pathway for documentation, escalation, safety planning and continuity rather than leaving them to improvise after repeated incidents.

### 4.2. Abandonment Risk Should Be Treated as a Regulatory-Uncertainty Problem

A recurring concern is that a nurse who withdraws from an abusive patient relationship could face an allegation of abandonment or professional misconduct. This manuscript does not rely on a specific reported Alberta disciplinary decision in which an RN or NP was sanctioned solely for ending an abusive relationship. No such directly analogous public decision was identified in the sources reviewed. The concern is instead a regulatory-risk and clarity concern: where the regulator has not provided a dedicated standard, nurses may reasonably fear that their decision will later be judged through broad duties to provide care, continuity expectations, employer policies, or case-by-case interpretations.

CNPS legal-risk materials illustrate this uncertainty. The CNPS states that terminating nursing services should be viewed as a last resort and that nurses may wonder whether discontinuing services is suitable where boundary violations, aggressive or threatening behaviour, or lack of response are present [27]. The CNPS also advises attention to documentation, client needs, alternative services and continuity when nursing services are discontinued [27,28]. These are prudent safeguards, but they are not the same as a regulator-issued Alberta standard. Legal-risk advice can help a nurse after uncertainty has arisen; a professional standard should reduce the uncertainty in advance.

The absence of a published disciplinary case should not be interpreted as adequate protection. Complaints may be resolved privately, through advice, undertakings, employer processes or risk-management decisions that never become reported decisions. More importantly, nurses should not have to test the boundary of abandonment through a complaint process before knowing what their regulator expects. A clear standard would protect the public precisely because it would define the difference between unsafe abandonment and justified, documented, non-discriminatory termination or restriction with continuity safeguards.

### 4.3. Patient Rights, Vulnerable Populations and Safeguards Against Misuse

Any standard that permits termination or restriction must guard against misuse. Patients with a mental illness, cognitive impairment, substance use disorders, trauma histories, communication barriers, disability, poverty, homelessness, language barriers, limited health literacy, or prior experiences of discrimination may behave in ways that are distressing, dysregulated or difficult to manage. Indigenous patients and other racialized or socially marginalized communities may also have legitimate mistrust of health systems because of historical and ongoing harms. A nurse-specific standard must distinguish vulnerability and complexity from abuse and safety risk.

The key safeguard is that termination should be conduct-based, not status-based. A nurse should not terminate or restrict a relationship because a patient is mentally ill, cognitively impaired, uses substances, is homeless, is Indigenous, is racialized, has a disability, refuses advice respectfully, complains about care, requires accommodation, or has complex needs. The relevant question should be whether the patient’s or family member’s actual conduct creates a credible safety risk, substantially damages the therapeutic relationship, or prevents safe, competent and ethical care after reasonable alternatives have been considered, except where urgency makes alternatives unsafe.

Safeguards should include human-rights screening, consideration of reasonable accommodation, trauma-informed communication, cultural safety, interpreter use, involvement of a support person or substitute decision-maker where appropriate, capacity and clinical-risk assessment, de-escalation, team review, chaperones, changed communication channels, transfer within a team, employer involvement, and written continuity planning. The standard should also preserve complaint rights and explain that termination cannot be retaliatory. These safeguards protect patients and public trust while still recognizing that vulnerability does not require an individual nurse to endure credible danger, sexual harassment, racist abuse, repeated intimidation or severe boundary violations.

## 5. Proposed Alberta Nurse-Specific Standard

### 5.1. Regulatory Form and Scope

The preferred reform is a CRNA-adopted standard or practice standard that applies to RNs and NPs. It should be written for all relevant settings: office-based nursing practice, nurse-led clinics, self-employed RN practice, NP primary care, community and home care, public-health services, virtual care, occupational health, long-term care and team-based programs. The standard should use both ‘ending’ and ‘restricting’ because full termination is not always necessary. Restriction may include transferring care to another provider, requiring appointments in a staffed setting, requiring a chaperone, limiting electronic communication to one monitored channel, using a clinic manager or case manager as an intermediary, or refusing home visits unless safety conditions are met.

The standard should also clarify whose relationship is ending. In some cases, only the individual nurse–patient relationship should end while the patient remains with the clinic or program. In other cases, the conduct may create a risk to all staff and justify discharge from the practice, subject to safeguards. In team-based or employer-run settings, the standard should require coordination with managers, professional-practice leaders, risk management, security and other clinicians. In self-employed practice, it should explain records custody, outstanding tests, referrals, privacy, prescriptions within scope and emergency instructions.

### 5.2. Permissible and Prohibited Grounds

Permissible grounds should include credible threats; physical aggression or attempted assault; repeated verbal abuse after expectations have been communicated where safe; sexual harassment or sexualized conduct; racist, discriminatory or degrading abuse directed at the nurse, staff, other patients or the public; stalking or harassment through electronic communication; refusal to respect professional boundaries; intimidation; behaviour by a family member, substitute decision-maker or visitor that creates comparable risk; and conduct that makes safe assessment or care impossible.

Prohibited grounds should be equally explicit. A relationship must not be ended or restricted because of race, ethnicity, Indigenous identity, colour, ancestry, place of origin, religion, sex, gender identity or expression, sexual orientation, marital or family status, age, disability, mental illness, medical complexity alone, substance use alone, poverty, homelessness, source of income, language, citizenship, respectful refusal of recommended care, request for a second opinion, filing of a complaint, or need for reasonable accommodation. Administrative inconvenience, personal dislike or the nurse’s disagreement with lawful patient choices should not be sufficient grounds.

### 5.3. Ordinary Pathway

For non-immediate situations, the standard should require a staged pathway. First, the nurse documents the behaviour objectively, including dates, words used where relevant, witnesses, safety concerns, clinical impact and steps already taken. Second, where safe, the nurse communicates behavioural expectations to the patient or family member in plain language. Third, the nurse considers alternatives such as de-escalation, accommodation, interpreter services, a support person, a chaperone, team transfer, appointment restrictions, virtual/in-person changes or communication limits. Fourth, the nurse consults the employer, clinical supervisor, professional-practice lead, risk management, the CNPS, a regulator practice consultant or legal counsel as appropriate. Fifth, if termination remains justified, written notice is provided with a timeline proportionate to the patient’s clinical needs and local access realities. Sixth, the nurse arranges continuity, including urgent follow-up, prescription bridging where within scope, test-result review, referrals, records transfer and emergency-care instructions.

### 5.4. Immediate Safety Pathway

The standard must also contain an urgent safety pathway with language as clear as the CPSA model. A registrant should be permitted to end or restrict direct contact immediately when the patient or a family member poses a credible safety risk; is threatening, violent or abusive; engages in sexual harassment, stalking or severe intimidation; uses racist or discriminatory abuse that threatens safety or dignity; refuses to respect professional boundaries in a way that makes direct care unsafe; or creates a situation in which the nurse cannot provide safe, competent and ethical care.

Immediate safety termination should not mean patient abandonment. It should mean that the individual nurse is not required to continue direct contact that exposes the nurse to credible harm or abuse. Continuity can be managed through safe channels: another clinician, a clinic manager, an employer, an administrator, a case manager, written notice, urgent-care instructions, emergency services, critical-result follow-up and records transfer. The standard should state that the nurse is not required to meet alone with the patient, speak directly with the patient, or continue abusive electronic communication where doing so would increase risk.

### 5.5. Documentation, Employer Integration and Practice Tools

Documentation should protect both parties. The record should include objective facts, clinical relevance, safety concerns, attempts to resolve the problem where safe, accommodations considered, consultations obtained, decision rationale, notice provided, continuity arrangements and records-transfer steps. Vague labels such as ‘difficult’ or ‘non-compliant’ should be avoided unless paired with specific facts. The standard should distinguish the clinical record, incident report, occupational health documentation and any privacy-sensitive safety information.

Employers and practice owners should integrate the standard into violence-prevention plans, incident reporting, home-visit risk screening, chaperone policies, communication policies, virtual-care procedures, security response, critical-result management and post-incident psychological support. Self-employed nurses should have a respectful-conduct policy, patient intake language, incident-log process, warning-letter template, termination-letter template, records-transfer process, urgent-care plan and protocol for abusive electronic communications. These should be treated as professional safety tools, not optional business conveniences.

Model language could read the following: ‘A registrant may end or restrict an established nurse–patient relationship when, after consideration of patient needs and available alternatives except in urgent circumstances, the patient’s or family member’s behaviour creates an unacceptable risk to the registrant, staff, other patients or the public; substantially damages the therapeutic relationship; or prevents the registrant from providing safe, competent and ethical care.’ The urgent provision should add the following: ‘Where credible safety concerns, threats, violence, abuse, stalking, sexual harassment, discriminatory abuse or unsafe boundary violations are present, the registrant may end or restrict direct contact immediately and may communicate through a safe intermediary or written channel. Reasonable steps must be taken to address urgent clinical needs, critical results, records transfer and emergency-care information.’ Table 2 presents a concise operational pathway for applying the proposed Alberta nurse-specific standard in ordinary and urgent safety situations.

## 6. Discussion

The analysis distinguishes governing authority from comparative policy values. The CPSA is the strongest jurisdictional comparator because it is an enforceable Alberta standard operating within the province’s professional-regulatory framework. CNO, BCCNM and CRNS materials are strong professional design comparators because they show how nursing regulators have operationalized discontinuation, documentation, continuity, abuse and safety. Although they do not govern CRNA registrants, they provide evidence of feasibility and regulatory convergence, as well as detailed components that can be transferred into Alberta policy. These sources perform different evidentiary functions; treating them as complementary avoids both dismissing nursing comparators because they are non-binding and treating external guidance as if it already supplied Alberta regulatory authority.

The CPSA provides the in-province precedent for a clearly defined immediate safety pathway, while the nursing comparators provide profession-specific procedural detail. An Alberta standard should not copy any one source mechanically. It should synthesize the strongest elements: permissible and prohibited grounds, reasonable efforts to resolve concerns where safe, documentation, written communication, continuity, employer and team integration, and immediate restriction or termination when direct care is unsafe.

The strongest objection is that a termination standard could be misused against vulnerable patients. This concern is legitimate. It is also a reason to regulate the process, not a reason to leave the process undefined. A clear standard can require objective documentation, accommodation analysis, cultural safety, trauma-informed practice, continuity planning and non-discrimination. Silence does not protect vulnerable patients; it can produce informal, inconsistent and poorly documented decisions. Clear standards protect patients by making the reasons, process and safeguards visible.

A second objection is that continuity may be difficult in rural, remote or underserved communities. This is also true. Scarcity of alternatives should influence the ordinary notice period, use of team-based transfer, involvement of health authorities, and interim care arrangements. However, scarcity should not force an individual nurse to remain in direct contact with a person who has created a credible risk of harm or repeated abuse. The standard should separate continuity of care from compulsory exposure of one nurse to danger.

A third objection is that workplace safety law already exists. It does, and it is essential. But workplace safety law is not the same as nursing professional regulation. A violence-prevention plan may tell an employer how to prevent or report hazards; it does not tell a self-employed RN, an NP in primary care, a home care nurse, or a clinic nurse how to end a continuing professional relationship without abandonment risk. The professional standard must fill that space.

Finally, a patient-centered approach does not require tolerance of abuse. Patient-centered care requires respect, dignity, access, continuity and fairness. It also requires safe conditions for the professionals providing care. Public trust is strengthened when the regulator makes both sides of the obligation clear: nurses must not abandon, discriminate against or retaliate against patients, and patients and family members cannot be allowed to coerce care through threats, intimidation, harassment or abuse.

## 7. Limitations and Future Research

This article has limitations. It is a narrative regulatory and policy perspective rather than an empirical study or systematic review. It relies on public documents and does not include confidential regulator files, employer incident data, complaint files, interviews, patient narratives or original survey data. No patient, service-user or public advisory group was involved in preparing the manuscript. The article also does not provide legal advice. Its recommendations would require legal, regulatory, Indigenous, patient, disability, rural, employer, union and public consultation before adoption.

A further limitation is that the article identifies a regulatory-risk problem without relying on a specific publicly reported Alberta disciplinary decision against a nurse for terminating an abusive patient relationship. This limitation has been made explicit. The absence of a reported case does not remove the need for a standard; it underscores that the argument is prospective and preventive. A standard should be created before nurses must learn its boundaries through complaints, fear or informal risk avoidance.

Future research should evaluate the prevalence of patient- and family-initiated abuse across nursing practice settings; nurses’ understanding of abandonment risk; how often abusive relationships are restricted or ended; complaint outcomes after termination or restriction; effects on nurse retention, moral distress and psychological safety; patient perceptions of fairness; rural and remote access consequences; equity impacts for Indigenous, racialized, disabled, unhoused and substance-using patients; and whether template letters, decision tools and employer integration improve safety and continuity. Implementation should be monitored with both safety and equity indicators.

## 8. Conclusions

The findings support protection for Alberta nurses that is comparable in regulatory clarity to that provided to Alberta physicians. A nurse’s duty to provide care is a serious professional obligation, but it is not a duty to endure threats, sexual harassment, racist or discriminatory abuse, stalking, intimidation, repeated boundary violations or unsafe direct contact. The relevant question is how nursing regulation can define a fair, documented and non-discriminatory pathway that prevents abandonment while allowing a nurse to end or restrict an abusive relationship.

The CPSA demonstrates that a regulator can combine an explicit immediate safety pathway with safeguards against arbitrary discharge. CNO, BCCNM and CRNS materials further demonstrate that nursing regulators can operationalize resolution efforts, documentation, continuity, transfer and abuse-related discontinuation. Taken together, these findings support an Alberta nurse-specific standard tailored to RNs and NPs across self-employed, office-based, community, home care and virtual practice. The standard should include permissible and prohibited grounds, ordinary and urgent safety pathways, documentation requirements, continuity safeguards, employer integration and practical templates.

A clearly defined regulatory pathway would align nurse safety with patient protection. Nurses cannot reliably provide safe, competent, ethical and compassionate care when they remain uncertain whether justified self-protection could be characterized as professional misconduct. Clear regulation would make care safer, fairer and more accountable for everyone involved.

## Figures and Tables

**Table 1:** Comparator findings and implications for an Alberta nursing standard.

Source or Framework	Key Contribution	Limitation for Alberta Nurses	Implication for Proposed Standard
CPSA physician standard	Expresses Alberta pathway; written notice; prohibited grounds; continuity; immediate discharge for safety risk, abuse or boundary violations.	Applies to physicians, not nurses.	Use as the central Alberta jurisdictional comparator for regulatory authority, process design and the immediate safety pathway.
CRNA Alberta nursing materials	Practice standards, NP FAQ, self-employed RN advice, documentation and unsafe-practice resources.	Important but fragmented; immediate-termination wording is not yet an all-registrant RN/NP standard.	Create a single standard for RNs and NPs across offices; communities; and virtual, home care and independent practice.
Criminal, OHS and human rights law	Address intimidation, workplace prevention duties, reporting, training, injury support and non-discrimination.	Do not define how to end or restrict an established nurse–patient relationship.	Integrate legal duties as safeguards, but do not rely on them as the full professional pathway.
Ontario, British Columbia and Saskatchewan nursing comparators	Demonstrate feasibility; provide operational detail, regulatory convergence and policy-transfer templates.	Do not govern CRNA registrants; vary by role, source type and explicitness of urgent authority.	Use as profession-specific design comparators alongside CPSA’s Alberta precedent.
International workplace-violence frameworks	Confirm global need for prevention, reporting, employer accountability and post-incident support.	Generally address workplace systems rather than relationship termination.	Support transferability and future research but not jurisdiction-specific Alberta protection.

**Table 2:** Concise operational pathway for an Alberta nurse-specific standard.

Step	Ordinary Pathway	Urgent Safety Pathway	Patient and Public Safeguards
1. Identify and triage	Assess conduct, clinical context, vulnerability and relationship impact.	Separate nurse from danger; seek assistance; call security or emergency services if needed.	Do not treat diagnosis, disability, poverty, culture or complaint-making as grounds.
2. Document	Record objective facts, witnesses, words used where relevant, safety risk and clinical impact.	Record immediate risk and why direct contact is unsafe or impracticable.	Avoid stigmatizing labels; protect privacy; distinguish clinical and incident records.
3. Attempt resolution where safe	Communicate expectations; consider accommodation, de-escalation, support person, interpreter, chaperone or team review.	Bypass direct discussion if it increases risk.	Use trauma-informed, culturally safe and non-discriminatory processes.
4. Restrict or transfer	Limit communication, require staffed appointments, transfer within team, or require chaperone.	Impose immediate no-direct-contact or safe-channel communication.	Preserve access to necessary care through safer alternatives where feasible.
5. End relationship	Provide written notice, reasons where safe, effective date, interim plan and records-transfer instructions.	End direct relationship immediately; notify through safe channel or intermediary.	Address urgent needs, critical results, medications within scope and emergency instructions.
6. Review and learn	Debrief, update care plan, assess recurrence and review employer policy.	Debrief promptly; assess psychological support and security measures.	Monitor equity, rural access, complaints, continuity outcomes and staff safety.

The pathway is intended to be adapted to Alberta legislation, CRNA standards, employer policy, practice settings and urgency of clinical needs.

## Data Availability

No new empirical datasets were generated or analyzed. All legal, regulatory and scholarly materials discussed in this perspective are publicly available and cited in the reference list.

## Public Involvement Statement

No members of the public, patients or service users were involved in the preparation of this perspective.

## Guidelines and Standards Statement

This manuscript is a narrative regulatory and policy perspective; no formal reporting guideline was applicable.

## Abbreviations

The following abbreviations are used in this manuscript:

Abbreviation	Meaning
ANA	American Nurses Association
BCCNM	British Columbia College of Nurses and Midwives
CNPS	Canadian Nurses Protective Society
CNO	College of Nurses of Ontario
CPSA	College of Physicians and Surgeons of Alberta
CRNA	College of Registered Nurses of Alberta
CRNS	College of Registered Nurses of Saskatchewan
NP	Nurse practitioner
OHS	Occupational health and safety
RN	Registered nurse
WPV	Workplace violence
